# Mine, Yours, Ours*?* Sharing Data on Human Genetic Variation

**DOI:** 10.1371/journal.pone.0037552

**Published:** 2012-06-05

**Authors:** Nicola Milia, Alessandra Congiu, Paolo Anagnostou, Francesco Montinaro, Marco Capocasa, Emanuele Sanna, Giovanni Destro Bisol

**Affiliations:** 1 Università di Roma “La Sapienza”, Dipartimento di Biologia Ambientale, Roma Italy; 2 Istituto Italiano di Antropologia, Roma, Italy; 3 Università di Cagliari, Dipartimento di Biologia Sperimentale, Cagliari, Italy; University of Florence, Italy

## Abstract

The achievement of a robust, effective and responsible form of data sharing is currently regarded as a priority for biological and bio-medical research. Empirical evaluations of data sharing may be regarded as an indispensable first step in the identification of critical aspects and the development of strategies aimed at increasing availability of research data for the scientific community as a whole. Research concerning human genetic variation represents a potential forerunner in the establishment of widespread sharing of primary datasets. However, no specific analysis has been conducted to date in order to ascertain whether the sharing of primary datasets is common-practice in this research field. To this aim, we analyzed a total of 543 mitochondrial and Y chromosomal datasets reported in 508 papers indexed in the Pubmed database from 2008 to 2011. A substantial portion of datasets (21.9%) was found to have been withheld, while neither strong editorial policies nor high impact factor proved to be effective in increasing the sharing rate beyond the current figure of 80.5%. Disaggregating datasets for research fields, we could observe a substantially lower sharing in medical than evolutionary and forensic genetics, more evident for whole mtDNA sequences (15.0% vs 99.6%). The low rate of positive responses to e-mail requests sent to corresponding authors of withheld datasets (28.6%) suggests that sharing should be regarded as a prerequisite for final paper acceptance, while making authors deposit their results in open online databases which provide data quality control seems to provide the best-practice standard. Finally, we estimated that 29.8% to 32.9% of total resources are used to generate withheld datasets, implying that an important portion of research funding does not produce shared knowledge. By making the scientific community and the public aware of this important aspect, we may help popularize a more effective culture of data sharing.

## Introduction

There is now wide consensus among researchers about the importance of achieving an effective, responsible and robust form of data sharing to advance scientific progress [1], [2]. From a historical point of view, the first people to mention the need to guarantee unrestricted availability of research data for scientific reuse was the American sociologist, Robert King Merton, in 1942. In his seminal essay “The Normative Structure of Science”, Merton included the common ownership of scientific discoveries (communalism) and the scrutiny for errors and inconsistencies to which all forms of knowledge should undergo (organized skepticism) among the main ethical principles of science [3]. A review of more recent scientific literature shows that the issue of data sharing has not been ignored in the last half century [4], [5]. However, it is only in the last two decades that it has become an explicit priority for biological and biomedical research [6], [7], due to the rapid increase in the production of data following the diffusion of computer-assisted technologies and digitalization techniques. Accordingly, various strategies have been set up to encourage researchers to share their results, including organization of *ad hoc* meetings and the development of explicit policies by scientific Journals and funding bodies [8], [9], while the setting up of primary online databases and repositories has provided permanent tools for data storage and dissemination [10], [11].

### Data sharing: opportunity or burden?

Some recent papers have discussed the *pros* associated with data sharing, pointing to the benefits of more rapid and efficient progress in research, better exploitation of data, optimized use of resources, opportunities for data quality control and promotion of scientific creativity [12], [13]. On the other hand, concerns have been raised regarding the actual spread of data sharing habits among researchers. In fact, it has been argued that the better exploitation of data and optimized use of resources may be counteracted by the time and economic costs required, not to mention underlying ethical concerns, and conflicts of interest with patenting discoveries [14]–[17]. In this contrasting scenario, empirical evaluations of data sharing may be regarded as an indispensable first step in the identification of critical aspects and the development of more effective strategies to increase availability of research data for the scientific community as a whole [18], [19].

### Sharing data on human genetic variation

Research concerning human genetic variation may be regarded as a potential forerunner in the establishment of a widespread sharing of primary datasets. This is possibly due to the codified nature of genetic information, the availability of infrastructures for data dissemination and the importance of research from the point of view of disease diagnosis, prevention and therapy [20], [21]. But, is data sharing common-practice in this research area? Despite the relevance of this subject, no specific analysis has yet been conducted. In fact, the most pertinent study carried out to date is a large scale US survey conducted in the broader field of genetics one decade ago. This investigation concluded that data withholding may limit some scientific activities, including attempts to analyze, replicate and compare published results [22].

Here we present the results of a study on data sharing in published studies of mitochondrial DNA (mtDNA) and Y-chromosome variation in human populations. These unilinear genetic systems are currently used in anthropological, forensic and medical research and applications [23]–[25]. They also provide a data basis for advanced computational approaches in population genetics [26], [27]. The relative homogeneity in terms of types of polymorphic variation makes mitochondrial and Y-chromosomal polymorphisms easily-handled sources of information for a pilot study of data sharing.

We analyzed the rate and type of data sharing in mitochondrial and Y-chromosomal datasets retrieved from papers indexed in the Pubmed database between 2008 and 2011, comparing different research fields and evaluating the effect of explicit editorial policies and impact factor rank. Based on the results obtained, we advance proposals on how to implement more effective data sharing policies and popularize the usefulness of data sharing throughout the scientific community and the public.

## Materials and Methods

**Figure 1:**
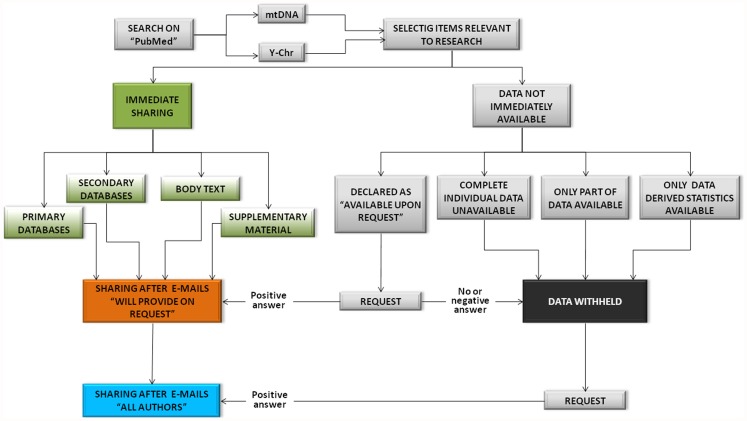
Procedure used to analyze data sharing in papers regarding human genetic variation. We retrieved a total of 1187 papers indexed between 1^st^ January 2008 and 31^st^ December 2011 in the PubMed using the key words “mtDNA human populations” and “Y chromosome human populations”. We set the following limits: “humans” for species and “English” for language. The procedure used for data request by email is described in figure 2. E-mails “will provide on request” were sent to the corresponding authors to request information from papers where data availability upon request is explicitly declared; E-mails “all authors” were sent to all corresponding authors who withheld datasets.

The initial dataset included 1187 papers indexed between 1st January 2008 and 31st December 2011 in the PubMed database (http://www.ncbi.nlm.nih.gov/pubmed), which were retrieved using the key words “mtDNA human populations” and “Y chromosome human populations” (see figure 1). After removing irrelevant studies (e.g. studies not pertinent to human populations, reviews or meta-analyses), a total of 253 mitochondrial and 290 Y-chromosomal datasets was extracted from 508 papers that had been published in 101 different Journals (see Table S1 for a brief description of datasets under scrutiny). The raw data file is available as (Table S2).

**Figure 2:**
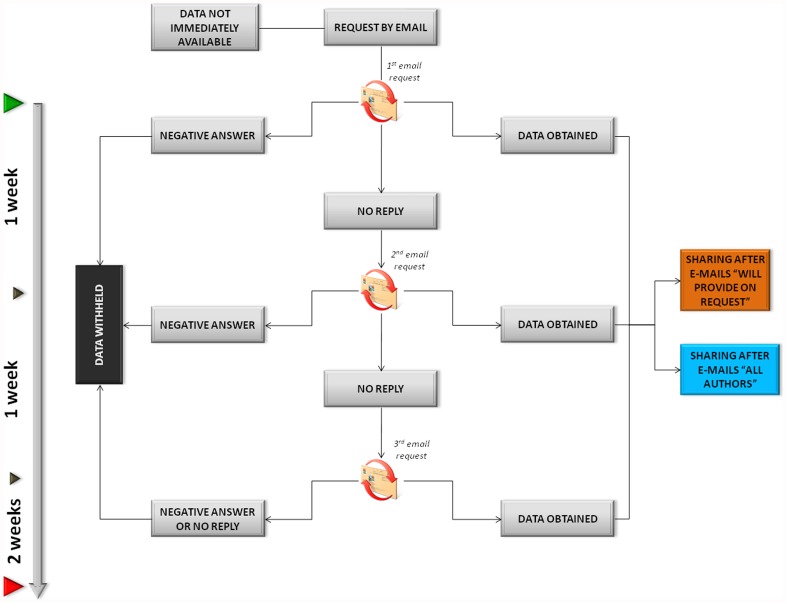
Procedure used to request data from corresponding authors of withheld datasets. The first two e-mails were sent by the first author (nicola.milia@uniroma1.it) of this paper, while the third one was sent by the corresponding author (destrobisol@uniroma1.it). The data collection was closed five weeks after the first request. E-mails “will provide on request” were sent to the corresponding authors of papers where data availability upon request is explicitly declared; E-mails “all authors” were sent to all other corresponding authors who withheld datasets.

Datasets were analyzed using the procedure described in the flowchart reported in figure 1. Only datasets reporting full information, which can be analyzed without any form of limitation, were counted as sharing. On the other hand, datasets lacking haplotypic information or were incomplete (e.g. which make only a part of raw data produced fully available or present only data-derived statistics) were included in the “withholding” categories (see Table S3 for more details on datasets categorized as withheld). We split our classification into shared or withheld dataset according to the information contained in the corresponding papers, trying to recover missing data from databases or repositories only when they were explicitly indicated in the text. As a complement to the examination of published papers (from which we obtained the “immediate sharing” rate), we asked corresponding authors of withheld datasets (including both authors declaring data availability upon request and others not giving any indication) to send missing information. This was done through 3 sequential requests which were e-mailed over a three-week period (Figure 2). In order to avoid any influence on author response, we made no mention of our study of data sharing in these messages (see Text S1).

The shared and withheld datasets were analyzed in relation to: (i) the research field to which the study may be assigned; (ii) type of editorial policy of the publishing Journal; (iii) impact factor rank of the publishing Journal; (iv) number of citations received; (v) approximate quantity of resources used to generate the datasets. In all these analyses, we considered as shared both datasets shared immediately and after e-mails sent to authors of papers declaring data availability upon request.

Datasets were divided into evolutionary, medical and forensic fields. All these three research fields study genetic and genomic differences within and among populations, but can be distinguished according to their final objectives. Essentially, we assigned papers (and the corresponding datasets) concerned with the evolutionary history of human groups, mainly in terms of demography and adaptation, or with the evolutionary processing acting on the human genome to Human Evolutionary Genetics. Papers dealing with the identification of individuals or test of parentage relationships for legal purposes were allocated to the Forensic Genetics field. Finally, we allotted publications concerned with causes and inheritance of genetic disorders, as well as with their diagnosis and management to Medical Genetics. When the assignment of a given paper to more than one field of research seemed to be possible or research aims were ambiguous or not explicit, the ISI category of the scientific journal was used as an additional criterion.

The type of editorial policy was rated using the information provided in the guide to authors of each journal: weak editorial policies are those where the authors are invited to share data, whereas in strong policies, data sharing is indicated as mandatory (see ref. 9 for a more detailed analysis of journal policies). Impact factor ranks were based on impact factor values released by ISI Reuters in June 2009.

We also determined the number of citations received by shared and withheld datasets and estimated the proportion of resources used to generate the data analyzed here. Citations were counted using the Scopus database (http://www.scopus.com). In order to make data comparable, each citation was weighted by considering the number of months passed since the publication of the cited paper. Very recent papers (published in the last six months of 2011) and self-citations from all authors were excluded from this analysis. To disentangle the effect of various variables which could potentially influence the number of citations, a multivariate analysis was carried out using a linear regression approach with the impact factor, time since publication and number of authors as covariates. Following Piwowar et al. 2007 [13], the number of citations and impact factor were log transformed.

In order to obtain an approximate estimate of resources used for the production of shared and withheld datasets, we first defined the parameter “Cost unit” (CU) for each type of mitochondrial and Y-chromosomal polymorphism. Essentially, adopted CU values are based on the number of sequencer runs needed to generate the corresponding data (Table S4). We considered two different CU values for complete mtDNA sequencing, mtDNA SNP and Y-chromosome SNP genotyping since their cost may vary substantially depending on the method used. The approximate cost for each dataset was obtained by multiplying the cost unit/s of the polymorphism/s analyzed by the number of individuals actually genotyped for each polymorphism. In these calculations, we assumed that data sharing does not imply any additional cost. In fact, depositing data in most of the online databases for mtDNA and Y-chromosome polymorphisms (e.g. GenBank, YHRD and EMPOP, see below) is completely free. Furthermore, nothing is usually paid to publishers for supplementary online material.

A file (in access format; File S1) which makes it possible to carry out a step by step reproduction of our protocol is provided as supplementary material.

## Results and Discussion

Our study focuses on human genetic variation, a research area that has yet to be studied despite its primary importance in the context of scientific data sharing. We based our approach on three main methodological choices. First, we retrieved the datasets to be inspected using a key-word driven search in Pubmed (see [28] for a similar approach), the largest public database of published research biomedical papers, rather than focusing on specific Journals [18], [29], [30]. In this way, we could better evaluate the overall situation in studies of human genetic variation and in specific research fields (Evolutionary, Forensic and Medical Genetics). Second, we tried to overcome the simple shared/withheld distinction, by better defining the various ways in which data are shared or withheld. This makes it possible not only to assess the ease of access to genetic information but also to better define the ways of presenting data which do not permit any effective sharing. Third, we complemented the inspection of published papers with serial requests to the authors of withheld datasets in order to obtain a more realistic estimate of the actual availability of data for scientific reuse.

### Data sharing is not yet common-practice in studies of human genetic variation

**Figure 3:**
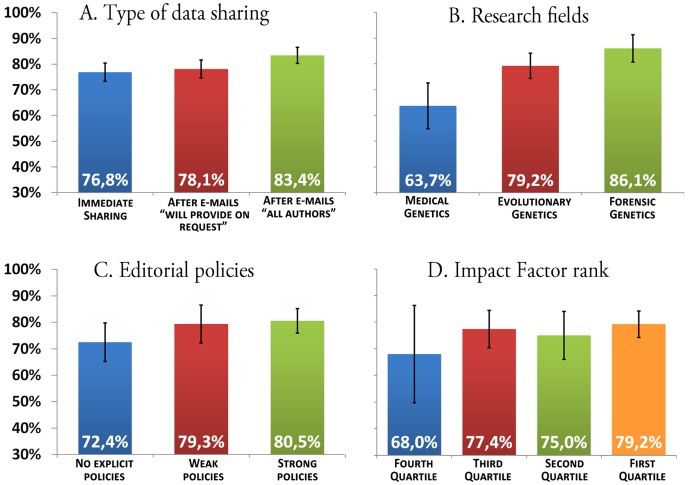
Sharing rates in published datasets regarding human genetic variation. Vertical bars indicate 95% confidence intervals. Separate results for mtDNA and Y chromosome polymorphisms are reported in Table 1. (A) In the “Immediate sharing” category, we reported the rate of datasets shared in the main text, its supplementary material or online databases which were explicitly indicated in the paper; E-mails “will provide on request” were sent to the corresponding authors to request information from papers where data availability upon request is explicitly declared; E-mails “all authors” were sent to all corresponding authors who withheld datasets. The results reported in frames B, C and D were obtained using the sharing rates obtained including the positive answers to E-mails “will provide on request”. We considered as negative the responses where authors asked for detailed information about the use of datasets and/or requested coauthorship before sending data. (B) Datasets were assigned to each research field according to the research aims, as stated in the paper. When assignment of a given paper to more than one field of research seemed to be possible or research aims were ambiguous or not explicit, the ISI category of the scientific Journal was used as an additional criterion. (C) The type of editorial policy was rated using the information provided in the guide to authors: weak editorial policies are those where the authors are invited to share data, whereas in strong policies, data sharing is indicated as mandatory. (D) Ranks were based on impact factor values released by ISI Reuters in June 2009.

We show in figure 3 that a substantial proportion of datasets (23.2%) is not immediately shared through the published material or information contained therein (body text, supplementary material or online databases), while an important fraction (16.6%) continues to be withheld even after serial e-mail requests to all authors of withheld datasets. No significant difference was observed between mitochondrial and Y-chromosomal polymorphisms (Table 1), but the relatively frequent use of GenBank for mtDNA data (69 out of 185 shared datasets, corresponding to 37.3%) makes them more easily downloadable (Table 2). Most withholding is due to the fact that results are presented only as data-derived statistics (75 out of 119 datasets, 63.0%), whereas less frequently, individual data are presented but they are not available in complete form (10 out of 119, 8.4%) or only a data subset is actually shared (34 out of 119, 28.6%) (see Table S3 for more details).

**Table 1:** Data sharing rates in studies of genetic variation of mitochondrial and Y-chromosomal variation in human populations.

	*mtDNA*	*Y chromosome*
***A. Type of data sharing***		
immediate sharing	73.1% (185/253)	80.0% (232/290)
sharing after e-mails “will provide on request”, cumulative value	73.1% (185/253)	82.4% (239/290)
sharing after e-mails “all authors”, cumulative value	76.7% (194/253)	89.0% (259/290)
***B. Research field***		
Human Evolutionary Genetics	79.7% (98/123)	78.9% (112/142)
Forensic Genetics	89.6% (43/48)	84.6% (99/117)
Medical Genetics	53.6% (44/82)	90.3% (28/31)
***C. Editorial policies***		
no explicit policy	63.5% (40/63)	79.3% (65/82)
weak policies	70.0% (42/60)	88.5% (54/61)
strong policies	79.2% (103/130)	81.6% (120/147)
***D. Impact Factor rank***		
Fourth quartile	61.5% (8/13)	75.0% (9/12)
Third quartile	80.0% (52/65)	75.0% (54/72)
Second quartile	60.0% (24/40)	87.5% (42/48)
First quartile	74.2% (95/128)	84.2% (107/127)

Mitochondrial and Y chromosomal datasets published in the same paper were analyzed separately. E-mails “will provide on request” were sent to request data from the corresponding authors of papers where data availability upon request is explicitly declared; E-mails “all authors” were sent to all corresponding authors of withheld datasets. Unless specified, values refer to the sharing rate observed after e-mails “will provide on request”.

**Table 2:** Types of data sharing (absolute values) in the examined dataset.

	*immediate sharing*					*sharing after* e-mails “will provide on request”*		*sharing after* e-mails “all authors”**	
	*GenBank*	*secondary*	*body text*	*supplementary material*	*tot.*		*tot.*		*tot.*
		*databases*							
**mtDNA**	69	0	81	82	185	0	185	9	194
**Y chr.**	0	0	106	127	232	7	239	19	258

* E-mails “will provide on request” were sent to the corresponding authors to request information from papers where data availability upon request is explicitly declared.

** E-mails “all authors” were sent to all corresponding authors of withheld datasets.

Only nine papers declaring availability upon request were found, which makes any evaluation of the rate of positive responses to e-mail requests very preliminary. Nonetheless, it should be noted that not all of the corresponding authors (7 out of 9; 77.8%) actually sent their primary datasets. As expected, a significantly lower rate of positive responses was obtained from corresponding authors of the remaining withheld datasets (29 out of 117, 24.8%). Our overall rate of positive responses (36 out of 126; 28.6%) is higher than reported in a previous study carried out using papers published in PLoS Clinical Trials and PLoS Medicine (1 out of 10, 10%) [18] but not far from what observed in Journals published by the American Psychological Association (64 out of 249, 25.7%) [30].

### There is a substantial variation in sharing rate of primary datasets across distinct research fields

We observed significantly lower sharing rates in Medical Genetics than in Human Evolutionary Genetics and Forensics (figure 3B). The value for Medical Genetics actually conceals a marked difference between maternally and paternally inherited polymorphisms (53.6% and 90.3%, respectively; see Table 1). Interestingly, only a 15.0% sharing rate was observed for complete mtDNA sequences (263 out of 1752 sequences), the most highly informative mitochondrial datasets. By contrast, most of this type of data (2719 out of 2730, 99.6%) is made available in evolutionary and forensic studies.

### Adoption of explicit editorial policies or impact factor rank has a limited effect on data sharing rates

A slightly higher sharing rate was observed for datasets published in journals with strong editorial policies and high impact factor rank (figure 3C and 3D, respectively), a result consistent with the positive association between the policy strength and data sharing that had been previously observed in a study of gene expression microarray data [9]. However, no difference between classes for each parameter is statistically significant, considering both the total and partial datasets (Figure 3 and Table 1). Furthermore, neither factor was associated with a sharing rate beyond 80.5% in the entire dataset (figure 3C, D). As previously observed [9], impact factor ranks and editorial policies were found to be significantly associated (p<0.001; Chi-square test for R×C contingency tables).

Our multivariate analysis showed that time since publication and impact factor are the main factor influencing the number of citations received by datasets (see Table S5). A slight increase (8.9%) in the number of citations was observed for shared datasets, with a more pronounced advantage (20.6%) for mtDNA (Table S6), but, again, no difference was found to be associated with a statistically significant result in our multivariate analysis.

### Some evidence-based proposals on how to increase data sharing in studies of human genetic variation

As a logical development of our study, after data analysis we focused on the implications of our results for the implementation of more effective data sharing strategies. The evidence-based proposals discussed here may complement recommendations of wider significance [31].

The substantially lower sharing rate observed for Medical rather than for Evolutionary and Forensic Genetics suggests that the type and/or impact of factors limiting data sharing may vary even among closely related fields of research. This finding points to the need to set up tailored approaches for each research field to more effectively increase overall data sharing. Potential conflicts with privacy issues, and/or lack of awareness of medical researchers regarding the usefulness of data (especially from control groups) for other research fields may account for this important difference. Other potential explanations are discussed in a recent study of raw gene expression microarray datasets, where it has been shown that authors of studies on cancer and human subjects were least likely to make their datasets available [32]. The author of this paper suggests that perceiving the cancer research field as being highly competitive and having connections with industry may combine with privacy issues and make researchers less willing to share their data. The first two conditions are probably more present in medical than in forensic or evolutionary genetic research.

The rate of positive responses by corresponding authors to our e-mails requesting primary datasets was higher than experienced in previous studies [18], [30]. However, even in our case, a large portion of requested datasets (90 out of 126; 71.4%) remains withheld after serial e-mails. The difficulties in recovering withheld data after their publication imply that complete and effective data sharing should be viewed in editorial policies as a requisite to be fulfilled before the paper is finally accepted for publication, rather than a simple recommendation.

Among the numerous editorial policies we scrutinized, those of *International Journal of Legal Medicine* (IJLM) and *Forensic Science International Genetics* (FSIG) may be taken as a model [33], [34]. Authors submitting papers to these two journals must first send their data to the Y Chromosome Haplotype Reference Database (www.yhrd.org) and European mtDNA Population Database (http://empop.org/) [35], [36]. After data quality control is passed, papers are subjected to peer review. In cases of final acceptance, data must be presented as individual haplotypes, usually as an electronic supplement. We observed that the sharing rates of datasets published in IJLM and FSIG (89 out of 99, 89.9%) contribute to the lower level of data withholding we observed for forensic compared to evolutionary and medical genetics (see above). Therefore, the editorial policies of these two journals may have a substantial impact on the availability of high-quality forensic data. The fact that they seems to be not 100% effective seems to reflect a widespread difficulty in obtaining the respect of editorial policy by the authors. As shown by a recent study carried out on a selection of 500 studies published in the 50 research journals with the highest impact factor, 30% of papers were not subject to any data availability policy, but an even higher percentage (58%) did not adhere to the existing data sharing guidelines [19].

It is also important to note that scientific journals may benefit from adopting stringent sharing data rules since papers whose datasets are available without restrictions are more likely to be cited than withheld ones (see above and ref. 13). Naturally, this may help increase their impact factor, and IJLM and FSIG are indeed the Journals with the highest impact factor in their category “Medicine, Legal” of the Science Citation Index (release 2010).

Availability of online databases which permit data downloading is a factor which does not directly affect data sharing but may have an impact on the ease of access to the data, especially for large datasets. We observed that an important part of information is shared through online databases for mtDNA but not for Y-chromosomal polymorphisms. It is worth noting in this respect that while there is only one scientifically curated online population database for Y chromosome data (Y chromosome Haplotype Reference Database, YHRD), several alternatives are available for mtDNA polymorphisms (e.g. EMPOP, Mitomap and GenBank).

Finally, through our study, we came to realize that there is an important aspect which could help popularize a more effective culture of data sharing among young researchers and throughout the whole scientific community. In fact, we show that a significant part of resources could be better exploited for research in human genetic variation if data sharing were to become more widespread.

By means of an approximate calculation method (see Table S2), we estimated that 29.8% to 32.9% of the total resources employed in the production of experimental data analyzed here were used to generate withheld datasets, with a noticeable difference between mitochondrial and Y-chromosomal data (37.1%–38.5% for mtDNA; 21.8%–26.9% for Y-chromosome). Interestingly, these ranges exceed the percentage of withheld datasets (26.9% for mtDNA and 17.6% for Y-chromosome). A box plot graph shows a slightly larger proportion of outliers among withheld than shared datasets (see Figure S1 for further details). After removing outliers from calculations, the range estimate of relative cost of withheld datasets returns closer to their relative percentage (from to 22.8% to 28.8% for mtDNA and 16.1% to 21.8% for Y-chromosome). This indicates that a minority of large-scale withholding papers has further decreased the ratio between benefits (information available to the scientific community) and costs (resources employed) of human genetic variation studies.

In conclusion, our study provides evidence that the majority of published data regarding human genetic variation are made openly available to the scientific community. However, we also show that further efforts are still needed to make data sharing common-practice in this research area. We argue that human genetic variation research could really become a forerunner for the establishment of widespread data sharing by making editorial policies more stringent, adapting strategies to the features of each specific research field and popularizing the advantages of data sharing in terms of optimized use of resources. On a more general note, we hope that the present study could pave the way for further investigations in other areas of genetic and biological research. In this sense, the simple data analysis protocol presented here could offer a useful reference and a common basis for future empirical studies of data sharing.

## Supporting Information

Figure S1 **Boxplots showing the distribution of Cost Unit values for shared and withheld datasets.** A slightly higher proportion of outliers was consistently observed for withheld datasets both for pooled (14.3% vs 9.7% and 15.1% vs 11.1% for low and high cost sets, respectively) and disaggregated data (17.6% vs 8.1% and 14.7% vs 9.2% for mtDNA; 15.7% vs 9.7% and 11.8% vs 10.5% for Y chromosome).
(TIF)

File S1 **Access file implementing the protocol for the analysis of data sharing.**
(MDB)

Text S1 **E-mail text used to request datasets to corresponding authors.**
(DOC)

Table S1 **Characterization of the datasets under scrutiny in terms of genetic polymorphisms.**
(DOC)

Table S2 **Mitochondrial and Y chromosomal datasets used in this study.**
(XLS)

Table S3 **Types of data withholding (absolute numbers) in the examined dataset.**
(DOC)

Table S4 **Cost units (CUs) adopted to obtain an approximate estimate of resources used for the production of datasets.**
(DOC)

Table S5 **Multivariate analysis of citations received by shared datasets.**
(DOC)

Table S6 **Citations received by shared and withheld datasets, as reported in the Scopus database (** http://www.scopus.com/home.url **; accessed on 02/03/2012).** In order to make data comparable, each citation was weighted by the number of months passed since the publication of the cited paper. It was not possible to retrieve the citations for 2 mitochondrial datasets due to the absence of the corresponding papers in the Scopus database.
(DOC)
