# Supplementary figures and images for: Mine, Yours, Ours? Sharing Data on Human Genetic Variation
Source: PLoS One. 2012 Jun 5;7(6):e37552. doi: 10.1371/journal.pone.0037552 (PMC3367958; doi:10.1371/journal.pone.0037552)

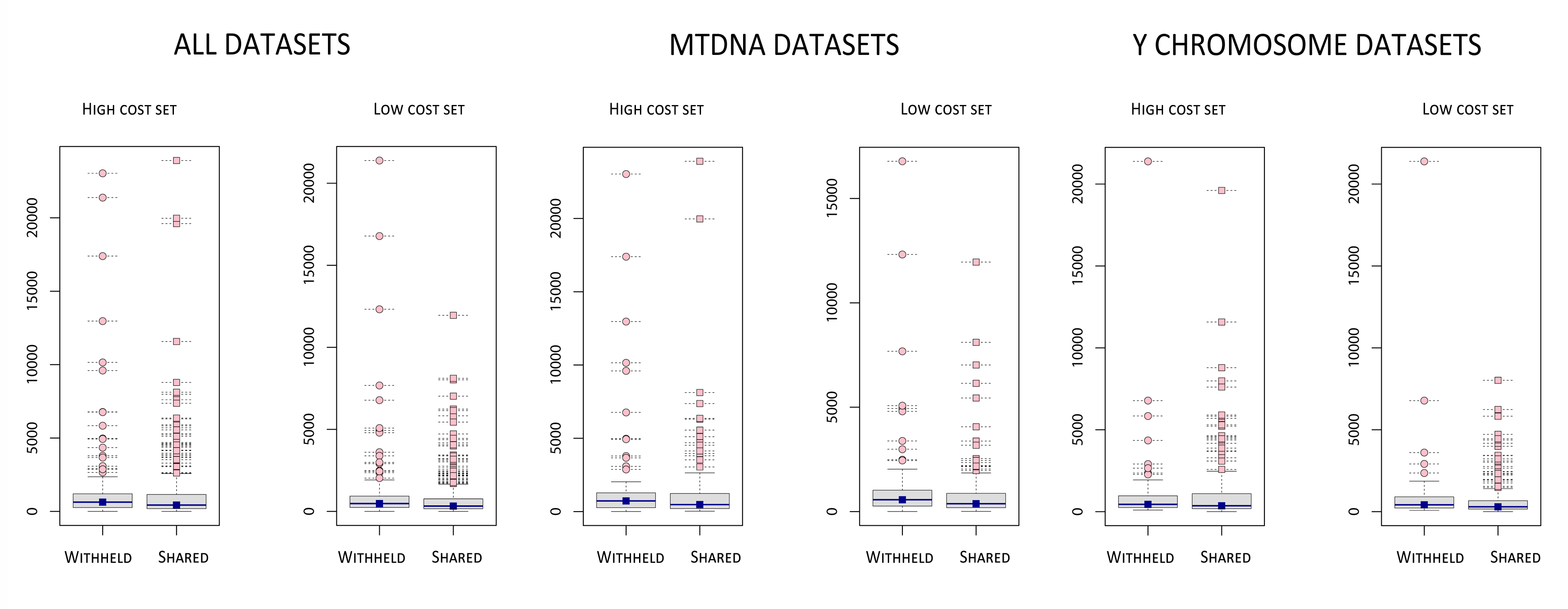

Supplement: Figure S1 — Boxplots showing the distribution of Cost Unit values for shared and withheld datasets. A slightly higher proportion of outliers was consistently observed for withheld datasets both for pooled (14.3% vs 9.7% and 15.1% vs 11.1% for low and high cost sets, respectively) and disaggregated data (17.6% vs 8.1% and 14.7% vs 9.2% for mtDNA; 15.7% vs 9.7% and 11.8% vs 10.5% for Y chromosome). (TIF) [file pone.0037552.s001.tif]
