# Supplementary material for: Mine, Yours, Ours? Sharing Data on Human Genetic Variation
Source: PLoS One. 2012 Jun 5;7(6):e37552. doi: 10.1371/journal.pone.0037552 (PMC3367958; doi:10.1371/journal.pone.0037552)
Supplement: Table S1 — Characterization of the datasets under scrutiny in terms of genetic polymorphisms. (DOC) [file pone.0037552.s004.doc]

**Table S1. Characterization of the datasets under scrutiny in terms of genetic polymorphisms.**

| **mtDNA (N=253)** | | | | | **Y-chromosome (N=290)** | | | |
| --- | --- | --- | --- | --- | --- | --- | --- | --- |
| control region sequence^a^ | SNPs^b^ | control region sequence and SNPs | complete genome^c^ | others^d^ | microsatellites^e^ | SNPs^b^ | SNPs and microsatellites | others^d^ |
| 48 (18.97%) | 43 (16.99%) | 92 (36.36%) | 63 (24.90%) | 7 (2.76%) | 124 (42.75%) | 54 (18.62%) | 108 (37.24%) | 4 (1.37%) |

^a^ Includes datasets that contains at least one sequence of complete or partial HVR I or HVR II regions.

^b^ Includes datasets that contains only information on single nucleotide polymorphisms.

^c^ Includes datasets that contains at least one complete mithocondrial genome.

^d^ Includes datasets that contains all the other types of information except the ones reported earlier (e.g. single gene sequences, ins/del, minisatellites etc..).

^e^ Includes datasets that contain only information on microsatellites loci.
