# Supplementary material for: Mine, Yours, Ours? Sharing Data on Human Genetic Variation
Source: PLoS One. 2012 Jun 5;7(6):e37552. doi: 10.1371/journal.pone.0037552 (PMC3367958; doi:10.1371/journal.pone.0037552)
Supplement: Table S3 — Types of data withholding (absolute numbers) in the examined dataset. (DOC) [file pone.0037552.s006.doc]

**Table S3. Types of data withholding (absolute numbers) in the examined dataset.**

|  | **Complete individual data unavailable*** | **Only part of data available**** | **Only data derived statistics available***** | **Total** |
| --- | --- | --- | --- | --- |
|  |
| **mtDNA** | 10 | 14 | 44^a^ | 68 |
| **Y chromosome** | 0 | 20 | 31^a^ | 51 |
| **Total** | 10 | 34 | 73 | 119 |

* More than one type of data was analyzed in the same individuals (e.g. STRs and SNPs for Y-chromosome or HVR-1 sequences and SNPs for mtDNA) but they are shared separately without cross references, so that it is not possible to reconstruct the entire individual genetic profile.

** Only a part of the data produced was made available; e.g. not all individual data and/or genetic markers are shared.

^a^ Includes the two datasets which were not retrieved after e-mails “Will provide on request”.
