# Supplementary material for: Mine, Yours, Ours? Sharing Data on Human Genetic Variation
Source: PLoS One. 2012 Jun 5;7(6):e37552. doi: 10.1371/journal.pone.0037552 (PMC3367958; doi:10.1371/journal.pone.0037552)
Supplement: Table S4 — Cost units (CUs) adopted to obtain an approximate estimate of resources used for the production of datasets. (DOC) [file pone.0037552.s007.doc]

**Table S4**. Cost units (CUs) adopted to obtain an approximate estimate of resources used for the production of datasets.

Values reported are based on the number of sequencer runs needed to generate the corresponding data. We applied two different values for complete mtDNA sequencing, mtDNA SNPs and Y-chromosome SNPs genotyping since their cost may vary substantially depending upon the method used (see below). The approximate cost for each dataset was obtained by multiplying the cost unit/s of the polymorphism/s analyzed by the number of individuals actually genotyped for each polymorphism (hierarchical genotyping strategies were considered only when it was explicitly stated in the text).

|  | **mtDNA** | | | | **Y-chromosome** | | |
| --- | --- | --- | --- | --- | --- | --- | --- |
|  | **partial or complete Control region** | **gene sequence** | **complete genome** | **SNPs** | **STRs** | **SNPs** | **1 Kb sequence** |
| lower CUs | 1 | 1 | 12 | 1 (up to 48 SNPs)^a^ | 1 (up to 17 STRs)^b^ | 1 (up to 48 SNPs)^c^ | 1 |
| upper CUs | 1 | 1 | 24 | 1 (up to 12 SNPs)^c^ | 1 (up to 17 STRs)^b^ | 1 (up to 12 SNPs)^c^ | 1 |

^a^ Each 48 or less SNPs were counted as 1: e.g. from 1 to 48 SNPs=1 CU; from 49 to 96 SNPs= 2 CUs.

^b^ Each 17 or less STRs were counted as 1: e.g. from 1 to 17 STRs=1 CU; from 18 to 34 STRs=2 CUs.

^c^ Each 12 or less SNPs were counted as 1: e.g. from 1 to 12 Snps=1 CU; from 13 to 24 SNPs=2 CUs.
