# Supplementary material for: Mine, Yours, Ours? Sharing Data on Human Genetic Variation
Source: PLoS One. 2012 Jun 5;7(6):e37552. doi: 10.1371/journal.pone.0037552 (PMC3367958; doi:10.1371/journal.pone.0037552)
Supplement: Table S5 — Multivariate analysis of citations received by shared datasets. (DOC) [file pone.0037552.s008.doc]

**Table S5**. Multivariate analysis of citations received by shared datasets.

|  | **Coefficient** | **Standard Error** | **t Stat** | **P-value** | **Lower 95%** | **Upper 95%** |
| --- | --- | --- | --- | --- | --- | --- |
| Intercept | -0,383344645 | 0,088728226 | -4,320436259 | 1,99558E-05 | -0,557810349 | -0,208878942 |
| Months since publication | 0,019153245 | 0,001279828 | 14,96547951 | 4,71722E-40 | 0,016636728 | 0,021669763 |
| Impact factor | 0,589504493 | 0,063703544 | 9,253872827 | 1,66832E-18 | 0,464244651 | 0,714764335 |
| Data Sharing | 0,007720118 | 0,039633662 | 0,194786904 | 0,84566498 | -0,070211281 | 0,085651518 |
| Number of authors | 0,01263145 | 0,0036388 | 3,47132317 | 0,00057807 | 0,005476503 | 0,019786397 |
