# Supplementary material for: Mine, Yours, Ours? Sharing Data on Human Genetic Variation
Source: PLoS One. 2012 Jun 5;7(6):e37552. doi: 10.1371/journal.pone.0037552 (PMC3367958; doi:10.1371/journal.pone.0037552)
Supplement: Table S6 — Citations received by shared and withheld datasets, as reported in the Scopus database ( http://www.scopus.com/home.url ; accessed on 02/03/2012). In order to make data comparable, each citation was weighted by the number of months passed since the publication of the cited paper. It was not possible to retrieve the citations for 2 mitochondrial datasets due to the absence of the corresponding papers in the Scopus database. (DOC) [file pone.0037552.s009.doc]

**Table S6.** Citations received by shared and withheld datasets, as reported in the Scopus database (<http://www.scopus.com/home.url>; accessed on 02/03/2012 ). In order to make data comparable, each citation was weighted by the number of months passed since the publication of the cited paper. It was not possible to retrieve the citations for 2 mitochondrial datasets due to the absence of the corresponding papers in the Scopus database.

|  | **all datasets** | | **mtDNA** | | **Y-chromosome** | |
| --- | --- | --- | --- | --- | --- | --- |
| all citations | shared | withheld | shared | withheld | shared | withheld |
| **average** | 0,199 | 0,183 | 0,228 | 0,189 | 0,178 | 0,176 |
| **median** | 0,139 | 0,111 | 0,150 | 0,111 | 0,130 | 0,118 |
| **st. dev.** | 0,011 | 0,019 | 0,020 | 0,026 | 0,024 | 0,027 |
|  |  |  |  |  |  |  |
| without self citations | shared | withheld | shared | withheld | shared | withheld |
| **average** | 0,152 | 0,134 | 0,173 | 0,139 | 0,137 | 0,128 |
| **median** | 0,100 | 0,083 | 0,105 | 0,083 | 0,099 | 0,083 |
| **st. dev.** | 0,009 | 0,015 | 0,017 | 0,020 | 0,011 | 0,009 |

.
