# Supplementary material for: Mine, Yours, Ours? Sharing Data on Human Genetic Variation
Source: PLoS One. 2012 Jun 5;7(6):e37552. doi: 10.1371/journal.pone.0037552 (PMC3367958; doi:10.1371/journal.pone.0037552)
Supplement: Text S1 — E-mail text used to request datasets to corresponding authors. (DOC) [file pone.0037552.s003.doc]

**Text S1.** E-mail text used to request datasets to corresponding authors.

**First and second e-mail request**

Dear Prof. “............”

I am a student working in the lab of Giovanni Destro Bisol (University of Rome "La Sapienza" and Istituto Italiano di Antropologia, [http://www.isita](http://www.isita/)-org.com/isita/Research.htm).

I have read with interest your article “............”

Although you provide a very useful description of the results obtained, they are not available as individual results for “...........................”,

which would allow me to make a complete set of comparisons between your data and those I have recently obtained.

Therefore, I would be extremely grateful if you could send me the published data.

Many thanks in advance for your help and time

Nicola Milia

**Third e-mail request**

Dear colleague,

following e-mails previously sent by Nicola Milia who works in my laboratory, I would like to ask if you could send me the results for “type of loci” (e.g. individual genetic profiles) of the paper "……"

I would be particularly grateful if you could send me the data within “… date…”

Thanks in any case for your attention.

with best regards

Giovanni Destro Bisol

Associate Professor of Anthropology

-------------------------------------------------
Giovanni Destro Bisol
Universita' di Roma La Sapienza
Dip.to di Biologia Animale e dell'Uomo
sede di Antropologia
P.le A. Moro 5 - 00185 Roma
tel 0039 06 49912273
fax 0039 06 49912771
